# Time to dietary diversity of complementary feeding improvements and its associated factors among infants aged 6–12 months in Ethiopia: evidence from performance monitoring for action

**DOI:** 10.3389/fnut.2025.1451193

**Published:** 2025-01-24

**Authors:** Feyisa Shasho Bayisa, Teshome Demis Nimani, Samuel Demissie Darcho, Abainash Tekola

**Affiliations:** ^1^Department of Epidemiology and Biostatistics, School of Public Health, College of Medicine and Health Science, Haramaya University, Harar, Ethiopia; ^2^Department of Health Service and Policy Management, School of Public Health, College of Medicine and Health Science, Haramaya University, Harar, Ethiopia; ^3^Department of Reproductive Health and Nutrition, School of Public Health, College of Medicine and Health Science, Haramaya University, Harar, Ethiopia

**Keywords:** dietary diversity, improvements, infants, women, Ethiopia

## Abstract

**Background:**

Infant and Young Child Feeding significantly affect the health, development, and nutritional status of children under 2 years old, ultimately affecting their survival. The aim of this study is to determine the time to improvement in dietary diversity and associated factors in infants aged 6 to 12 months.

**Methods:**

The study used secondary data from the PMA Ethiopia longitudinal panel survey, involving pregnant women from January to March 2024. The data management and analysis were performed using Stata version 17. The Kaplan–Meier survival curve (KM) and the log-rank test method were implemented. A Cox proportional-hazard regression model was used to explore the association between independent variables and the outcome variable. The strength of the association was indicated by the adjusted hazard ratio (AHR) with a 95% confidence interval. The threshold of *p* < 0.05 was applied to determine the significance of an association.

**Results:**

The study found that the proportion of infants with improved dietary diversity aged 6–12 months was 22% (95% CI: 19.5, 25%). Factors associated with improved dietary diversity in infants aged 6 to 12 months were married women (AHR = 9.3, 95% CI = 1.19, 8.30), women with a secondary school (AHR = 1.9, 95% CI = 1.05, 3.51), women with technical and vocational (AHR = 2.0, 95% CI = 1.01, 4.05) and women with a university degree (AHR = 2.9, 95% CI = 1.51, 5.38). Moreover, women in the highest wealth quintile (AHR = 3.5, 95% CI = 1.31, 9.41), women visiting PNC (AHR = 1.7, 95% CI = 1.13, 2.62), women visiting ANC 1–3, and more than four times were (AHR = 2.4, 95% CI = 1.51, 3.74) and (AHR = 3.6, 95% CI = 2.28, 5.67) times higher for improving dietary diversity.

**Conclusion:**

The findings of this study showed that the proportion of dietary diversity improvement was 22%. Which is relatively low. Marital status, educational status, wealth index, PNC, and ANC visits were identified as statistically significant factors associated with dietary diversity improvements. It suggests that public health interventions should focus on enhancing maternal knowledge and promoting regular healthcare visits to mitigate malnutrition and improve infant health outcomes in Ethiopia.

## Introduction

The health, development, and nutritional status of children under 2 years of age are directly influenced by the diet of infants and young children, which in turn has an impact on child survival (1). For children 0–23 months old, improving IYCF practices is essential for improved development, health, and nutrition (1). The guidelines recommend that meals for children aged 6–23 months should be varied and evenly spaced to meet their nutritional and energy needs (1–3).

In the world, over 3.4 million children under the age of five pass away every year because of improper feeding practices. Of these, improper feeding practices during the first 2 years of life are linked to two-thirds of these deaths (4, 5). An estimated 149 million children under the age of five were considered stunted (too short for their age), 45 million were considered wasted (extremely thin for their height), and 37 million were overweight or obese in 2022 (6). Of infants 0–6 months old, 44% are exclusively breastfed. Very few children obtain safe and nutritionally sufficient complementary foods; in many nations, less than one-fourth of infants between the ages of 6 and 23 months fulfill the requirements for dietary diversity and age-appropriate feeding frequency (6, 7). The issue may stem from socioeconomic disparity and inadequate healthcare accessibility across different nations.

The prevalence of severe forms of malnutrition, like wasting and stunting, is highest in sub-Saharan Africa (8). In Ethiopia, malnutrition is still a serious public health issue (8). Over the past few years, a large number of studies on nutrition and health in Ethiopia have been carried out. According to these studies, Ethiopia has the highest rate of malnutrition in all of Sub-Saharan Africa. According to the 2019 UNICEF report, one in ten Ethiopian children under the age of five (about two million) and 45 percent of child fatalities under the age of five are linked to undernutrition. Stunting still affects more than 5.4 million Ethiopian children under the age of five (39 percent) (9). The 2019 Ethiopian Demographic Health Survey (EDHS) reports that malnutrition is linked to around 53% of deaths among children under five (10). One of the most important tactics for enhancing child survival and promoting healthy growth and development is the promotion and protection of appropriate baby and early child feeding (8). However, only a small percentage of infants between the ages of 6 and 23 months worldwide satisfy the necessary standards for dietary diversity, and less than one-fourth of them are fed a diet that is sufficiently nutritious (7, 11). In order to maintain appropriate growth and development during this crucial stage, the World Health Organization recommends that an infant receive the minimum dietary diversity (MDD) of at least five food groups out of eight (11, 12). Although many children cannot meet these criteria, these seven food groups were grains, roots, and tubers; legumes and nuts; dairy products (milk, yogurt, cheese); flesh foods (meat, fish, poultry, and liver/organ meats); eggs; and other fruits and vegetables. For children to grow and stay healthy to the best extent possible throughout the first 100 days of life, adequate nourishment is essential. This allows the children to grow to their maximum potential (12). Making investments in a child’s first 2 years of life can not only promote healthy growth in low-income nations where early childhood undernutrition is frequent, but also potentially break the cycle of intergenerational malnutrition (10, 11). Breastfeeding is no longer sufficient to supply a growing child’s nutritional needs after the age of 6 months; as a result, eating a sufficient and varied diet is essential (13). Child mortality and morbidity are significantly reduced when the core indicators of Infant and Young Child Feeding (IYCF) practices are implemented. These indicators include early initiation of breast milk within 1 h of birth, exclusive breastfeeding for children under 6 months of age, initiation of complementary feeding at 6 months of age, continuation of breastfeeding until 1 year of age, and consumption of iron-rich or iron-fortified foods (6, 14).

A number of studies have demonstrated that children who receive a minimum amount of nutritional diversity are less likely to be underweight and stunted (15). The study conducted in the Oromia region showed that the proportion of children with a good dietary diversity score was 40 (26.7%) (16), Dire Dawa, Ethiopia 24.4% (17), Amhara, Ethiopia 18.2% (18), SNNP, Ethiopia (19), and Somali region, Ethiopia 47.2% (20). A similar study in Ethiopia revealed that the proportion of inadequate minimum dietary diversity was 85.1% (21). There is a substantial correlation between the use of dietary diversity among children aged 6–23 months and several sociodemographic characteristics, such as maternal education, occupation, and family affluence, and community-level factors, such domicile and regional variance (4, 13, 16, 18, 21, 22). The health and nutritional status of Ethiopians have suffered greatly as a result of the fact that approximately 60% of dads, mothers, and other caregivers are uneducated, and just 23% have completed elementary school. The health and nutritional status of Ethiopians have suffered greatly as a result of the fact that approximately 60% of dads, mothers, and other caregivers are uneducated, and just 23% have completed elementary school (23). Furthermore, the majority of teenagers have developed attitudes that underlie poor nutrition by the time they are 13–17 years old. In this age bracket, only 11% of respondents think a child under five can survive or be breastfed solely, and 26% think a baby can start eating animal products at 6 months. The eating habits and attitudes of adults that they observe in their communities are absorbed by the teenagers (23).

The government of Ethiopia understands that combating malnutrition is critical to attaining sustainable development. Consequently, audacious measures were implemented in the health and other nutrition-focused domains to establish policies, initiatives, and extensive interventions aimed at considerably diminishing all types of malnourishment among the most susceptible populations, namely, young children, expectant mothers, and nursing mothers. However, undernutrition in women and children continues to be a serious issue that calls for multisector action (9). So in general previous research has shown that mothers who gave birth in a health facility, had no economic problems, and received breastfeeding counseling during pregnancy had a higher rate of exclusive breastfeeding. In addition, mothers with higher levels of education and postnatal check-ups were more likely to introduce complementary foods correctly (24). However, the full continuum of age-appropriate infant feeding practices and associated factors in Ethiopia is not well documented. Therefore, the purpose of this study was to determine when to enhance the dietary variety practices of newborns and to evaluate the factors linked to these improvements.

## Materials and methods

### Study setting

The Performance Monitoring for Action (PMA) Ethiopia panel survey was conducted in four large, predominantly agrarian regions (Tigray, Oromia, Amhara, and Southern Nations, Nationalities, and Peoples’ Region), one pastoralist region (Afar), and one urban region (Addis Ababa) of Ethiopia, comprising 90% of the total Ethiopian population. With a population of over 80% living in rural areas, Ethiopia will be the second most populated country in Africa in 2022 with an estimated total population of 123 million (25).

Three levels of healthcare delivery systems comprise the Ethiopian health tier system: primary, secondary, and tertiary level healthcare organizations. The first level of health systems is district (woreda) systems, which are made up of primary hospitals that serve 60,000–100,000 people, health centers that serve 15,000–25,000 people, and satellite health posts that serve 3,000–5,000 people and are connected to one another by a referral system. General hospitals serve a population of 1.1–1.5 million people at level two, and specialized hospitals serve 3.5–5 million people at level three (26). According to the Ethiopian MoH 2020–2021 report on health indicators, there are 17,699 health posts, 3,777 health centers, and 367 public hospitals in the country (27).

### Study design and duration

The study was conducted using secondary data from the PMA Ethiopia longitudinal panel survey, a community-based prospective cohort study, where pregnant women were enrolled at baseline and interviewed at 6 weeks, 6 months, and 1 year postpartum from March to June 2024. The survey was launched in 2018 with the aim of generating timely and actionable data on reproductive, maternal, and newborn health (RMNH) indicators in Ethiopia. The PMA-Ethiopia panel survey was conducted from 2019 to 2021, which is the first cohort of the project.

### Source population

The source population for this study was all infants whose mothers are enrolled in the 2019–2021 cohort of the PMA-Ethiopia project.

### Study population

The study population for this study was all alive infants with their mothers on completion of the 1 year postpartum 2019–2021 cohort of the PMA-Ethiopia project.

#### Inclusion criteria

Women who consented to follow-up until the end of the survey and completed the survey with alive children at the time of the one-year postpartum follow-up interview were included in this study.

#### Exclusion criteria

Women who refused follow-up/missed interviews at some point, died, or had no live births were excluded from this study.

### Data source

The PMA-Ethiopia survey was conducted in cooperation between Addis Ababa University (AAU) and the Johns Hopkins Bloomberg School of Public Health (JHSPH). Three distinct survey components make up PMA Ethiopia: an annual cross-sectional survey that is conducted nationwide, a panel survey that follows pregnant women for a year after giving birth, and two cohorts of women from 2019 to 2021 and 2021 to 2023. The 1-year panel survey of the first cohort of postpartum women will provide the data for this study.

### Sampling technique and procedure

In the four areas of Tigray, Amhara, Oromia, and SNNP, a two-stage cluster sampling technique was applied by stratifying rural and urban residences. In the Afar region and Addis Ababa, two-stage cluster sampling without stratification was employed under the assumption that there would be no distinction between urban and rural areas because Addis Ababa is an urban area and a majority of the Afar people live in pastoralist communities. Initially, 217 enumeration areas (EA) were chosen at random from each of the six regions. Subsequently, each EA had 35 randomly chosen households, and at the time of the survey, every woman in those households underwent screening to determine whether she was pregnant.

Among the 2,868 pregnant or recently postpartum women enrolled in cohort 1 baseline survey of the PMA-Ethiopia Project, 255, 250, and 320 women missed interviews at 6 weeks, 6 months, and 1 year postpartum, respectively. Because women who had non-live births (*n* = 258) were not interviewed during the follow-up interviews, 1785 women were included in the analysis of determining the prevalence of dietary diversity in infants and counseling on the GMP program for caregivers/parents. Finally, using a purposive sampling procedure, women who had received counseling on the GMP program during PNC visits (*n* = 873) were included to examine the role of counseling on growth monitoring and promotion during postnatal care in improving the optimal dietary practice of infants.

### Study variables

#### Dependent variable

Time to improvements in dietary diversity.

#### Independent variables

Maternal age, Household Wealth, Marital status, Parity, Region, Residence, Maternal education level, Place of delivery, PNC visits, ANC visits, nutritional counseling.

### Operational definitions

Dietary Diversity improved an infant who consumed five or more food groups from the eight food groups during the previous day (11, 12).

The eight food groups are as follows: breast milk; grains, roots, and tubers; Legumes and nuts; Dairy products (milk, yogurt, and cheese); Flesh foods (meat, fish, poultry, and liver/organ meats); Eggs; Vitamin –A rich fruits and vegetables; and other fruits and vegetables.

Dietary Diversity not improved an infant who consumed four or fewer food groups from the eight food groups during the previous day (11, 12).

### Data collection procedures

In 217 EAs, a census of 36,614 households was conducted between October and November 2019. Following a screening of 32,792 women aged 15 to 49, who reported being pregnant or having given birth within the previous 6 weeks, these women qualified for the panel study; 2,889 of them were found to be eligible, and 2,855 of them enrolled for the baseline survey. In addition to completing a baseline survey upon enrollment, each woman had to participate in follow-up interviews 6 weeks, 6 months, and 1 year postpartum. The panel’s purpose is to identify variations in health-related behaviors among populations that have been “exposed” to different elements of Ethiopia’s healthcare system. Data on dietary practices and PNC counseling used for this study were reported at six-month interview with 91% response rate and at 1 year interview with 87% response rate (28).

Resident enumerators conducted baseline interviews, collecting data on women’s sociodemographic characteristics, antenatal care, family planning history, pregnancy intentions, and gestational age. Information was gathered on postnatal care visits, the child’s 24-h food intake, the initiation of complementary feeding, and nutritional counseling. Women 5–9 weeks postpartum at enrollment were asked about labor and delivery experiences. Informed consent was obtained from all 2,868 pregnant or recently postpartum women before participation. Women who were pregnant at enrollment underwent baseline and 6-week postpartum interviews. The PMA-Ethiopia Panel Cohort 1 6-week follow-up survey included selected participants from the baseline survey groups. Eligible participants for the 6-week follow-up survey included women 5–9 weeks postpartum at baseline, women 0–4 weeks postpartum at baseline consenting to follow-up, and pregnant women at baseline consenting to follow-up. Two groups of women (*n* = 277 and *n* = 2,393) were studied based on their postpartum status. The 6-week follow-up survey was conducted at different times for the two groups: between September and December 2019 and March and September 2020. A total of 2,669 women completed the 6-week follow-up survey. Data on ANC characteristics, labor, delivery situations, psychological factors, and postpartum experiences were collected during the follow-up. Face-to-face interviews were conducted at the women’s homes, and all data were self-reported to minimize recall bias. Women’s sociodemographic status, such as age, education, parity, region, wealth, residence, and marital status, was collected during the baseline survey and matched with follow-up responses. Parity, a sociodemographic characteristic, did not include the index or most recent pregnancy in the data analysis (29).

Enumerators gathered data on MNH services, PNC, and newborn nutrition via surveys. Information was collected at 6-week, 6-month, and 1-year postpartum follow-ups. Respondents were asked about health checks for themselves or their children. Those who received health checks were considered to have received PNC. Women reporting PNC received child nutrition counseling about various topics. Counseling included guidance on diverse foods, animal-source foods, feeding frequency, and avoiding sugary beverages. Receiving any PNC counseling was confirmed by affirmative responses to counseling content questions (29).

#### Data management and analysis procedures

The data were originally downloaded from the Open Data Kit (ODK) aggregate server daily during the data collection period, and the data management and analysis were performed using Stata version 17. Participants applied further data cleaning procedures to detect and handle outliers, missing values, and inconsistencies in variables and to address censorship. The Kaplan–Meier survival curve (KM) and the log-rank test method were implemented to test the occurrence of differences in the probability of dietary diversity improvement between groups. The proportional hazard assumption was checked by the Schoenfeld residual test and was satisfied (the *p*-value of each variable ranges from 0.056 to 0.995, and the global test result was 0.538, which was insignificant). Multicollinearity was checked using variance inflation factors (the mean VIF was 3.8). Cox proportional-hazard regression explored associations between variables. The Cox-Snell residual test was used to check the model’s fitness. Factors with a *p*-value <0.25 in bivariable analysis were entered into multivariable analysis. The crude hazard ratio (CHR) and adjusted hazard ratio (AHR) with a 95% confidence interval (CI) were calculated to assess the strength of the association between independent variables and time to dietary diversity improvements. Variables with a *p*-value of 0.05 were deemed statistically significant for improving dietary diversity among 6 to 12-month-old infants.

### Ethics and consent

The PMA Ethiopia received ethical approval from Addis Ababa University, College of Health Sciences (AAU/CHS) (Ref: AAUMF 01–009) and the Johns Hopkins University Bloomberg School of Public Health (JHSPH) Institutional Review Board (FWA00000387). Informed written consent was secured from the mothers/caregivers. To ensure the confidentiality of patient information, patients’ names and addresses, such as phone numbers, were not recorded during data collection. The entire study process followed the relevant guidelines and regulations of the Declaration of Helsinki.

## Results

### Background characteristics of study participants

Mothers those aged under 25, comprise the largest group both in the improved (40.41%) and not improved (43.68%) categories. Marital status significantly affects outcomes, with married women being overwhelmingly predominant in both improved (99.48%) and not improved (94.56%) groups. School level also shows a stark difference, with a higher percentage of improving outcomes among mothers with higher education levels (diploma and above, 19.69%). Urban residence correlates with a higher improvement rate (67.88%) compared to rural areas (32.12%). Orthodox mothers tend to have higher percentages in both improved and not improved groups, although other religious affiliations also show significant numbers. The highest quintile shows the largest percentage of improvement (68.39%). Geographic location affects outcomes significantly, with Addis Ababa showing a high improvement rate (29.53%), while regions like Afar show much lower percentages (0.52%) (Table 1).

**Table 1:** Background characteristics of participants by improvements dietary diversity in Ethiopia.

Variables	Categories	Improved		Not improved	
		Frequency	Percentage	Frequency	Percentage
Sex of infants	Male	97	50.26	367	53.97
	Female	96	49.74	313	46.03
Age in years	<25	78	40.41	297	43.68
	25–29	54	27.98	172	25.29
	30–34	39	20.21	116	17.06
	> = 35	22	11.40	95	13.97
Current marital status	No married	1	0.52	37	5.44
	Married	92	99.48	643	94.56
School level	Never attended	22	11.40	203	29.85
	Primary	65	33.68	261	68.24
	Secondary	45	23.32	123	18.09
	Technical and vocational	23	11.92	42	6.18
	Diploma and above	38	19.69	51	7.50
Place of residence	Urban	131	67.88	368	54.12
	Rural	62	32.12	312	45.88
Mothers religion	Orthodox	88	45.60	331	48.68
	Muslims	48	24.87	189	27.79
	Protestant	55	28.50	150	22.06
	Others	2	1.04	10	1.47
Wealth index	Lowest quintile	6	3.11	65	9.56
	Lower quintile	13	6.74	76	11.18
	Middle quintile	19	9.84	92	13.53
	Higher quintile	23	11.92	131	19.26
	Highest quintile	132	68.39	316	46.47
Region	Addis Ababa	57	29.53	115	16.91
	Afar	1	0.52	19	2.79
	Amhara	33	17.10	178	26.18
	Oromia	43	22.28	211	31.03
	SNNP	59	30.57	157	23.09

### Maternal related characteristics

The majority (94.82%) of the women with less than four number of parity improved dietary for their babies. Women with four or more ANC visits (23.83%) and those who received nutritional counseling (49.74%) experience notable improvements (Table 2).

**Table 2:** Maternal related characteristics of infants aged 6–12 months in Ethiopia.

Variables	Categories	Improved		Not improved	
		Frequency	Percentage	Frequency	Percentage
Parity	<4	183	94.82	591	86.91
	> = 4	10	5.18	89	13.09
ANC follow up	Yes	93	48.19	170	25
	No	100	51.81	510	75
Number of ANC follow up	No visits	86	44.56	509	74.85
	1–3	61	31.61	124	18.24
	4 and above	46	23.83	47	6.91
PNC visits	Yes	123	63.73	302	44.41
	No	70	36.27	378	55.59
Nutritional counseling	Yes	96	49.74	233	34.26
	No	97	50.26	447	65.74

**Figure 1:**
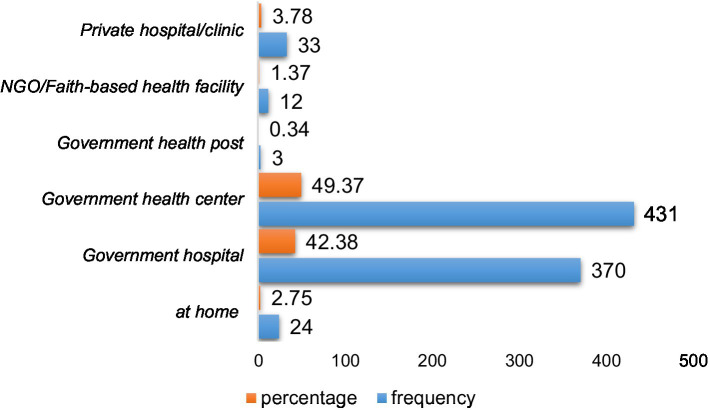
Proportion of infant’s delivery place.

The majority of births occurred at government health centers (49.37%), followed by government hospitals (42.38%). Only a small fraction of births took place at home (2.75%) (Figure 1).

**Figure 2:**
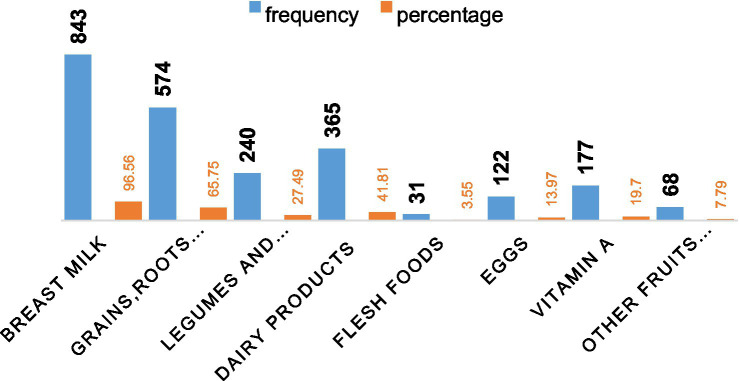
The percentage of infants took foods regularly in addition to breast milk after age of 6 months.

Nearly all 843 mothers (96.56%) nursed their babies from birth, according to the bar charts below. In addition to breast milk, the majority of the 574 newborns (65.75%) consumed the dietary classes of cereals, roots, and tubers. Flesh foods had the lowest recorded percentage of dietary diversity for babies 31 (3.55%) (Figure 2).

### Improvements of dietary diversity

**Figure 3:**
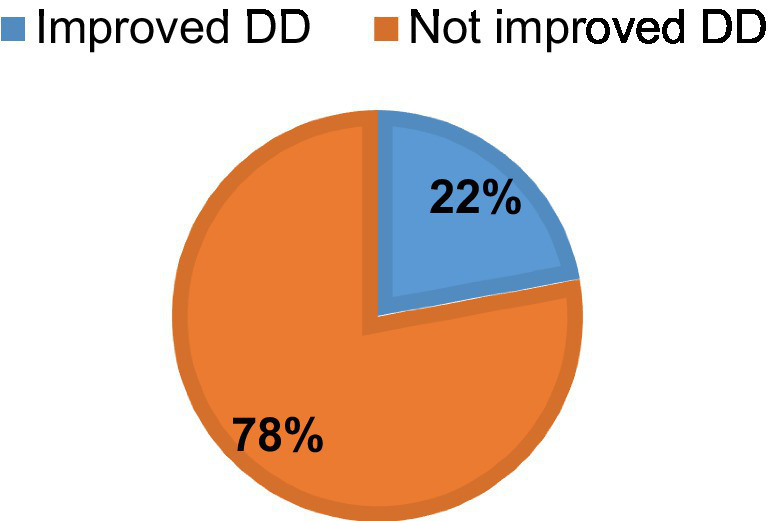
The percentage of improvements of dietary diversity among infants aged 6–12 months.

During the 6–12 month period, less than one in four newborns, 22% (95% CI: 19.5, 25%), consumed five or more food groups (Figure 3).

**Figure 4:**
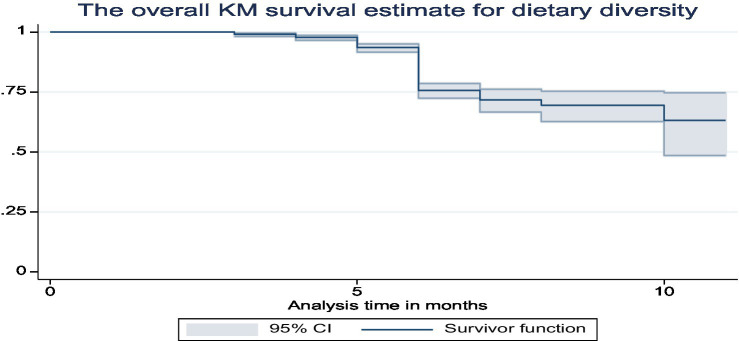
The overall Kaplan–Meier survival curve of dietary diversity among infants in Ethiopia.

The overall Kaplan–Meier survival curves show that the babies began consuming food other than breast milk when they were less than 6 months old (Figure 4).

**Figure 5:**
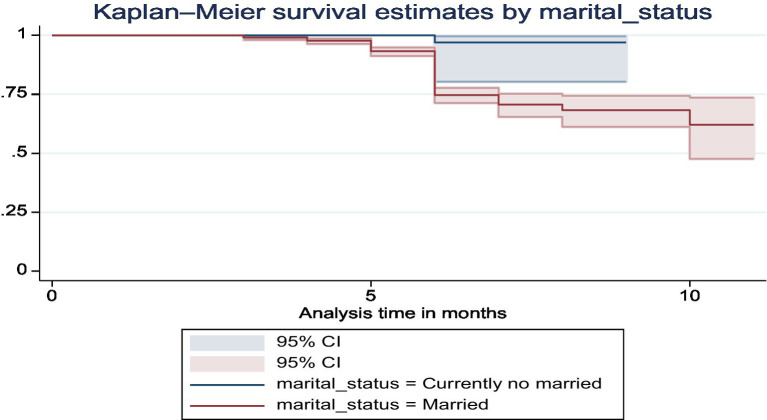
The Kaplan–Meier survival curve of dietary diversity among infants by marital status in Ethiopia.

The Kaplan–Meier curves demonstrated that married mothers greatly increased the dietary variety of their infants compared to single moms (chi-square value for log rank test = 8.58, *p* = 0.0034) (Figure 5).

**Figure 6:**
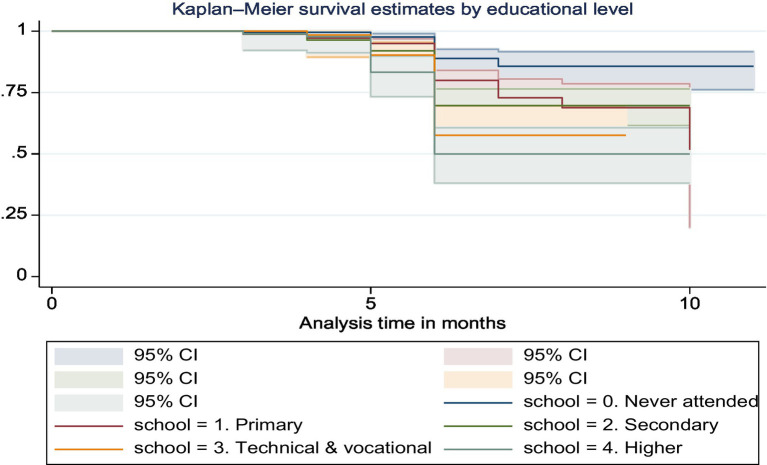
The Kaplan–Meier survival curve of dietary diversity among infants by educational status in Ethiopia.

According to the Kaplan–Meier curves, mothers who had completed formal education were far more likely to improve the nutritional diversity of their infants than those who had not (chi-square value for log rank test = 58.71, *p* = 0.0000) (Figure 6).

### Cox proportional hazard assumption test

The Schoenfeld residual test was used to evaluate the cox proportional hazard assumptions for each predictor’s variable. After the global test was run, the results showed that it was not significant (each variable’s *p*-value ranged from 0.056 to 0.995, and the global test as a whole was met with a *p*-value of 0.538) (Table 3).

**Table 3:** Schoenfeld residual test of dietary diversity improvements for infants aged 6–12 months in Ethiopia.

Variables	Rho	Chi2	DF	Prob > chi2
Region	−0.000	0.000	1	0.995
Current marital status	−0.027	0.140	1	0.708
School level	−0.028	0.150	1	0.696
Residence	0.009	0.010	1	0.918
Parity	0.017	0.060	1	0.810
Number of ANC follow up	−0.051	7.150	1	0.07
Delivery place	0.068	0.880	1	0.347
Nutritional counseling	−0.012	0.040	1	0.844
ANC visits	0.014	4.380	1	0.056
Wealth index	0.059	0.590	1	0.444
PNC visits	0.020	0.110	1	0.742
Global test	9.910		11	0.538

Rho (spearman correlation), DF (degree of freedom), Prob > chi2 (*p*-value).

**Figure 7:**
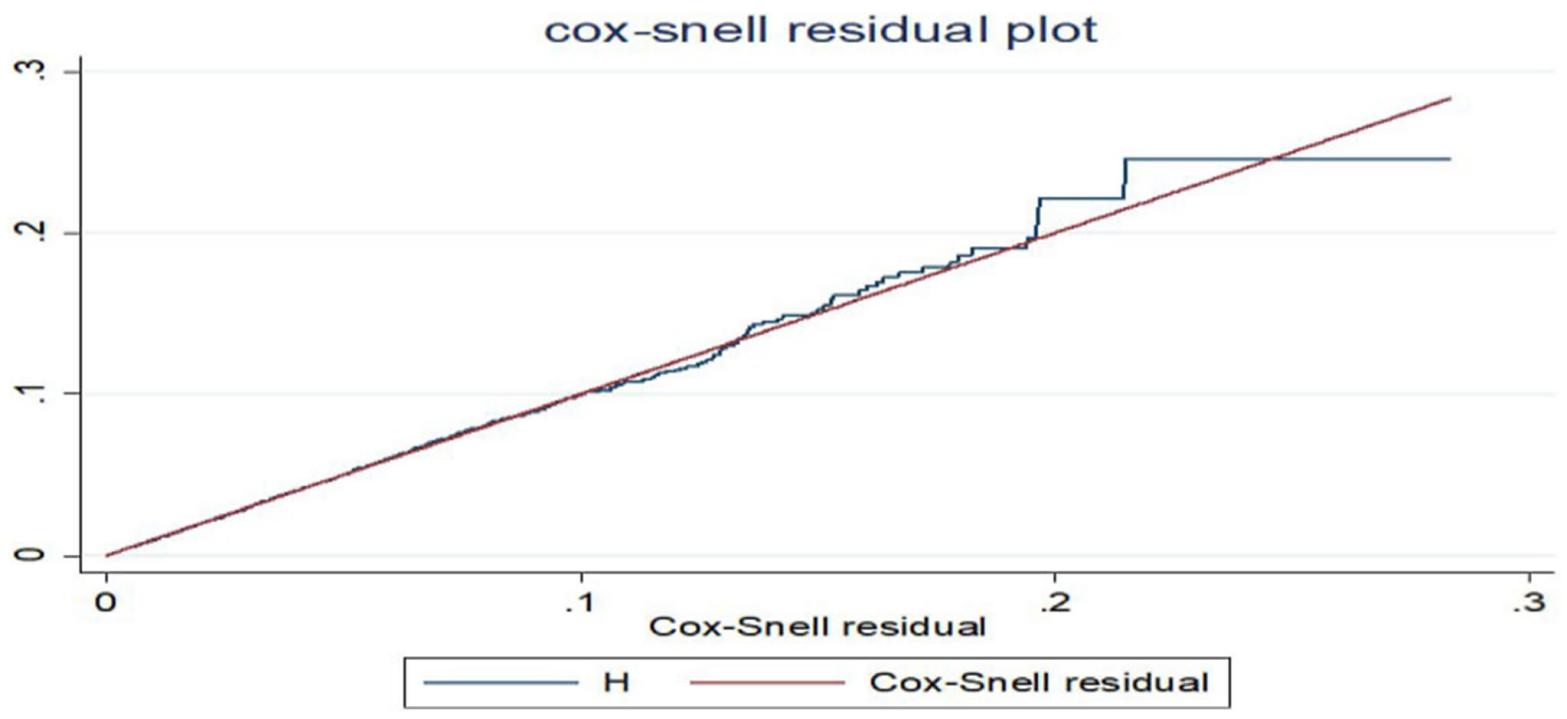
Cox–Snell residual plot of dietary diversity.

The hazard function closely follows the 45-degree line, as demonstrated by the Cox-Snell residual plot, indicating that the model’s goodness of fitness was met. The final model thus had good data fitting (Figure 7).

### Factors associated with dietary diversity improvement

All variables with a *p*-value of less than 0.25 in the bivariable Cox proportional hazards analysis were included in the multivariable Cox proportional hazards regression analysis. On this basis, marital status, education level, place of residence, wealth index, region, ANC visits, frequency of ANC follow-up, parity, PNC visits, nutritional counseling, and place of delivery were candidates for the multivariable Cox proportional hazards regression model. However, only five variables, such as marital status, education level, wealth index, PNC visits, and the number of ANC follow-ups, were statistically significant variables for dietary diversity. The improvement of dietary diversity in infants aged 6–12 months were 9.3 times higher in married women (AHR = 9.3, 95% CI = 1.29, 8.30). The likelihood of improving dietary diversity in infants aged 6–12 months was 1.9 times higher in women with a secondary school (AHR = 1.9, 95% CI = 1.05, 3.51). The probability of improved dietary diversity in infants aged 6–12 months was 2.0-fold (AHR = 2.0, 95% CI = 1.01, 4.05) and 2.9-fold (AHR = 2.9, 95% CI = 1.51, 5.38) higher in women with a technical and vocational, and higher degree, respectively, than in women without a school degree. Women in the highest wealth quintile were 3.5 times more likely (AHR = 3.5, 95% CI 1.31, 9.41) to have improved dietary diversity in infants aged 6–12 months than women in the lowest wealth index quintile. The likelihood of women visiting PNC were 1.7 times higher to improve dietary diversity for infants aged 6–12 months (AHR = 1.7, 95% CI = 1.13, 2.62). The likelihood of women visiting ANC 1–3 and more than four times were (AHR = 2.4, 95% CI = 1.51, 3.74) and (AHR = 3.6, 95% CI = 2.28, 5.67) times higher for improving dietary diversity for infants aged 6 to 12 months compared with no visits ANC (Table 4).

**Table 4:** Cox proportional hazards regression analysis for factors associated with dietary diversity improvement among infants 6–12 months in Ethiopia.

Variables	Categories	DD improvement		CHR (95%CI)	AHR (95%CI)
		Improved	Not improved		
Current marital status	No married	1	37	1	1
	Married	192	643	9.4 (1.32, 7.26)*	9.3 (1.29, 8.3)*
School level	Never attended	22	203	1	1
	Primary	65	261	1.9 (1.20, 3.12)*	1.5 (0.85, 2.54)
	Secondary	45	123	2.9 (1.71, 4.76)*	1.9 (1.05, 3.51)*
	Technical and vocational	23	42	3.9 (2.19, 7.07)*	2.0 (1.01, 4.05)*
	Diploma and above	38	51	5.3 (3.13, 8.98)*	2.9 (1.51, 5.38)*
Residence	Urban	131	368	1	1
	Rural	62	312	0.59 (0.44, 0.80)*	1.3 (0.77, 2.06)
Wealth index	Lowest quintile	6	65	1	1
	Lower quintile	13	76	1.7 (0.65, 4.52)	1.6 (0.60, 4.37)
	Middle quintile	19	92	1.9 (0.79, 4.93)	1.9 (0.76, 4.94)
	Higher quintile	23	131	1.8 (0.72, 4.35)	1.7 (0.65, 4.29)
	Highest quintile	132	316	3.8 (1.69, 8.70)*	3.5 (1.31, 9.41)*
Region	Afar	1	19	1	1
	Addis Ababa	57	115	8.6 (1.2, 2.03)*	3.7 (0.49, 2.29)
	Amhara	33	178	3.6 (0.49, 2.46)	2.3 (0.31, 1.84)
	Oromia	43	211	4.4 (0.61, 2.01)	3.1 (0.41, 2.57)
	SNNP	59	157	6 (0.83, 3.07)	3.5 (0.46, 2.56)
ANC visits	Yes	93	170	2 (1.48, 2.61)*	1.3 (0.84, 1.95)
	No	100	510	1	1
Parity	<4	183	591	1	1
	> = 4	10	89	0.40 (0.21, 0.76)*	0.74 (0.36, 1.54)
Nutritional counseling	No	97	447	1	1
	Yes	96	233	1.2 (0.92, 1.65)	1.1 (0.68, 1.55)
PNC visits	Yes	123	302	1.9 (1.44, 2.59)**	1.7 (1.13, 2.62)**
	No	70	378	1	1
Delivery place	At home	3	21	1	1
	At health facility	190	659	1.8 (0.59, 5.77)	0.86 (0.26, 2.86)
Number of ANC follow up	No visits	86	509	1	1
	1–3	61	124	2.1 (1.54, 2.97)**	2.4 (1.51, 3.74)**
	4 and above	46	46	3.5 (2.47, 5.08)**	3.6 (2.28, 5.67)**

DD, dietary diversity; CHR, crude hazard ratio; AHR, adjusted hazard ratio, *statistically significance at 0.05 and **statistically significance at 0.01 and 0.05; CI, confidence interval.

## Discussion

The World Strategy for Infant and Young Child feeding was created in collaboration with WHO and UNICEF to raise awareness of the effects that feeding habits have on newborns’ and children’s health, development, and survival. Based on research, the Global Strategy aims to promote optimal health outcomes by highlighting the critical role that adequate feeding habits play in nutrition during the early years of life. Infant and childhood morbidity and mortality are significantly increased by inadequate breastfeeding, particularly during the first 6 months of life, and are further exacerbated by inappropriate supplemental feeding (1). Therefore, the aim of this study was to identify the factors associated with dietary diversity improvements and timing to change dietary diversity in Ethiopia.

The study found that 22% (95% CI: 19.5, 25%) of infants aged 6–12 months experienced improvements in dietary diversity. This indicates that while some progress is being made, a significant portion of infants may still be consuming limited diets, which can have adverse effects on their growth and development. This finding highlights the need for continued efforts to enhance dietary diversity among infants. This finding is consistent with systematic study conducted in Ethiopia (23.25%) (30) and Kemba Woreda, Southern Ethiopia (23.3%) (31). However, the finding of the study is lower compared to the study conducted in Oromia region, Ethiopia (34.7%) (16), Wolaita zone, southern Ethiopia (43.2%) (32), Gedeo Zone, southern Ethiopia (29.9%) (19) Ashanti Region, Ghana (33.3%) (12), three West African countries (54.2%) in Côte d’Ivoire, (33.3%) in Niger and (30.8%) in Senegal (33). The variation may result from low purchasing power, food product affordability, and ignorance of the nutritional needs of babies and early children. This population also has distinct eating habits, with a tradition of preparing a limited variety of foods for the household. In addition, it seems that sharing food with siblings occurs frequently at home. The finding of the current study is also higher than study done in west Ethiopia (17.32%) (34), Southern Ethiopia (7.8%) (35), Northwest Ethiopia, rural Ghana (18.2%), Northwest Ethiopia (12.6%) (36), Ghana (15.3%) (37), India (8%) (38). The discrepancies may be due to the age difference as well as the fact that our study locations were urban area, where there may be a better understanding of a diverse diet. Recall, social desirability bias, and self-reported measures may also have an impact on the estimated dietary diversity.

Married women have a significant positive influence on dietary diversity improvements in infants. According to the study, infants whose mothers were married showed a higher likelihood of improved dietary diversity compared to those whose mothers were not married. The results highlight that out of 92 married women, 99.48% had improved dietary diversity for their infants, in contrast to only one non-married woman who had an improvement. The Kaplan–Meier survival curves provided further evidence, demonstrating that married mothers greatly increased the dietary variety of their infants compared to single moms (chi-square value for log-rank test = 8.58, *p* = 0.0034). This finding is similar with the study in Ethiopia (39). The association is consistent with other studies conducted in Ethiopia, indicating that married women are more likely to ensure better dietary diversity for their infants. The reasons behind this correlation could be multifaceted, including better household income stability, shared responsibilities, and possibly better access to nutritional information and support systems, aiding in providing a diverse diet for their children.

In the present study, infants whose mothers’ had secondary, technical and vocational, and higher educational status were more likely to improve dietary diversity which agreed with the findings of studies conducted in Ethiopia (10, 32, 36). The reasoning behind this correlation is that educated women tend to have a better understanding of the importance of dietary diversity for children. Higher educational status equips mothers with the knowledge regarding the nutritional needs of their infants, thus enabling them to provide a more varied and nutritious diet. Moreover, the Kaplan–Meier survival curve analysis further reinforces this finding, showing that mothers who completed formal education are significantly more likely to improve the nutritional diversity of their infants compared to those who did not, with a chi-square value for the log-rank test at 58.71 and a *p*-value of 0.0000.

The study found that infants from wealthier households exhibited higher dietary diversity. Women in the highest wealth quintile are found to be 3.5 times more likely to have improved dietary diversity in their infants compared to those in the lowest wealth quintile. This may be attributed to better access to a variety of food sources and the ability to purchase diverse foods. It suggests that economic factors play a crucial role in determining dietary options available to families. This finding is in line with several study conducted in Ethiopia (32, 40, 41). This correlation suggests that households with higher wealth indices tend to have better dietary diversity for their infants, probably due to increased food security and the ability to purchase a variety of foods. Therefore, addressing economic disparities and improving food security can be vital in enhancing dietary diversity for all infants, regardless of socioeconomic status.

The significant relationship between regular healthcare visits (both PNC and ANC) and dietary diversity improvements indicates that healthcare services are critical in promoting better feeding practices. The women who visits PNC were 1.7 more likely to improve the infant’s dietary diversity. This finding is supported the study done Ethiopia (30, 34, 41, 42). It could be because counseling on maternal and newborn feeding is one of the core activities at the PNC in our country, there is a positive impact on IYCF practice. Therefore, the content from the source suggests that healthcare workers providing nutrition counseling about balanced diets and appropriate Infant and Young Child Feeding (IYCF) practices during Postnatal Care (PNC) visits can positively affect dietary diversity improvements in infants. Pregnant women who visited ANC multiple times had a higher likelihood of improving the dietary diversity for their babies compared to those who did not visit ANC. This is consistent with findings from studies conducted in Ethiopia (37, 39, 42). This improvement is likely because antenatal care services enhance maternal counseling and community conversation programs on child feeding practices, thereby increasing mothers’ understanding of how to prepare and feed their children.

### Strengths and limitations

The study benefits from a large sample size and includes a diverse range of participants from different geographic regions, socioeconomic backgrounds, and maternal education levels, enhancing the generalizability of the findings. The study on infant dietary diversity, based on secondary data, lacks crucial variables like cultural practice, nutrition knowledge, and access to diverse food sources. Since dietary diversity is often assessed through self-reported data from mothers regarding their infants’ food consumption over the previous day, there is a risk of recall bias. Mothers may forget or misreport the types and quantities of foods consumed, leading to inaccuracies in dietary diversity assessments. Participants may provide responses that they believe are more socially acceptable or favorable, particularly regarding dietary practices. This can lead to over reporting of dietary diversity or adherence to recommended feeding practices.

## Conclusion

The study underscores the significance of determining the timing and identifying factors associated with dietary diversity improvements among infants in Ethiopia. The proportion of dietary diversity improvement among infants aged 6–12 months was 22%. Higher levels of maternal education were significantly associated with better dietary diversity. Infants from wealthier households exhibited higher dietary diversity. Regular healthcare visits were strongly linked to dietary diversity improvements. There is a need for increased awareness and education among mothers about the importance of dietary diversity for infants. The findings suggest that public health interventions should focus on enhancing maternal education, improving access to healthcare, and addressing economic barriers to food access. Programs that provide nutrition counseling during antenatal and postnatal care can be particularly effective in promoting better dietary practices among mothers, ultimately benefiting infant health. The study highlights that improving dietary diversity is not solely a matter of individual behavior but is influenced by broader socioeconomic and healthcare factors. Therefore, comprehensive strategies that integrate education, healthcare access, and economic support are essential for effectively addressing malnutrition and improving dietary diversity among infants. Establishing a robust system for monitoring dietary diversity and nutritional outcomes among infants can help identify gaps and inform future interventions. Researchers should consider conducting longitudinal studies to track dietary diversity changes over time and understand the long-term impacts of various interventions on infant nutrition. Incorporating qualitative research methods can provide deeper insights into the cultural beliefs, practices, and barriers that affect dietary diversity among infants. This can help tailor interventions to specific community needs.

## Data Availability

The raw data supporting the conclusions of this article will be made available by the authors, without undue reservation.
